# Knowledge on First Aid Management of Traumatic Dental Injuries Among Primary School Teachers of Dharan, Nepal

**DOI:** 10.1155/ijod/6614216

**Published:** 2025-12-17

**Authors:** Ashish Shrestha, Tarakant Bhagat, Santosh Kumari Agrawal, Ashma Ojha

**Affiliations:** ^1^ Department of Public Health Dentistry, B.P. Koirala Institute of Health Sciences, Dharan, Nepal, bpkihs.edu

**Keywords:** dental injuries, first-aid, knowledge, school teacher

## Abstract

**Introduction:**

Majority of the traumatic dental injuries (TDIs) occur at schools. School teachers are the immediate person to come in contact after dental injuries, so it is necessary for them to have knowledge about the first aid management of the injured tooth.

**Objective:**

To determine the knowledge of first aid management of TDIs among primary school teachers of Dharan, Nepal.

**Methods:**

A cross‐sectional questionnaire based study was conducted among primary school teachers of Dharan city. A self‐administered questionnaire was distributed among 110 school teachers equally from both public (*n* = 55) and private (*n* = 55) schools. Descriptive statistics, chi‐square test, and logistic regression analysis were used to report the data. The level of significance was set at *p*  < 0.05.

**Results:**

The mean age of the school teachers participating in the study was 36.38 ± 10.98 years. Majority of them (73.6%) were females. Only few school teachers (23.6%) were informed or trained about dental trauma. The primary source of information was through the internet (57.7%). More than two‐third of the school teachers had poor knowledge (85.5%) on first‐aid management of TDIs. A statistically significant difference was observed in the primary management of avulsed tooth between teachers of public and private schools (*p* = 0.007).

**Conclusion:**

The findings of this study concluded that primary school teachers of Dharan city had poor knowledge on first‐aid management of TDIs which reflects a need to improve their knowledge on the same.

## 1. Introduction

Traumatic dental injuries (TDIs) account for 5% of all body injuries, which occur frequently in children and young adults [1]. Twenty‐five percent of all school‐going children experience dental trauma with most affected age‐group being 7–12 years. Majority of TDIs (71%–92%) take place before the age of 19 years. Luxation injuries are most common TDIs in primary dentition, whereas uncomplicated crown fractures are the most common injuries occurring in permanent dentition [1, 2]. Overall, TDIs in a primary dentition (mean age of 3.4 years) have a global prevalence of 22.7%, and TDIs in a permanent dentition (mean age 13.8 years) have a global prevalence of 15.2%. The global incidence rate of TDI is 2.82 events per 100 persons per year with a mean age being 7.8 years. The global prevalence ratio of TDI in male to female is 1.43 suggestive of males being 35%–52% more susceptible to TDIs as compared to females. TDIs are reported to be highest in South‐East Asian regions [3].

About 60%–70% of all the TDIs involve maxillary incisors in both primary and permanent dentitions [2]. TDIs in primary dentition can have negative impact on erupting permanent dentition like tooth malformation, impacted teeth, and eruption disturbances [1]. Major etiologies of TDIs are considered to be due to falls, traffic or automobile accidents, strike with objects, sports injuries, or child abuse [2]. In addition to these, factors like increased overjet, incompetent lips, presence of illness (epilepsy, cerebral palsy, and hearing/visual impairment), inappropriate use of teeth, or iatrogenic causes increase the risk of TDIs [2, 4]. These injuries can have impact on day to day activities like feeling embarrassed to smile, laugh and show teeth, difficulty in social relationships, and inability to maintain healthy emotional state. Also, problems with eating and enjoying food, cleaning mouth, speaking, and pronouncing words clearly were found to be higher in children with TDIs [5]. A study done in Brazil by Bendo et al. [6] also reported that children with untreated TDI were 1.2‐fold more likely to feel upset and avoid smiling/laughing as compared to children without TDIs.

The majority of TDIs occur in and around home and schools. Hence, an immediate person who comes in contact after the injuries is either parents or school teachers. A significant association was also observed between the number of teeth traumatized and the place of occurrence of dental trauma where school was the most common incidence site for multiple trauma [7].

TDIs cause sufferings to the patients; since there can be bleeding from the soft tissues, teeth can be fractured, displaced, or lost; also, it causes parents or guardians to be anxious due to the traumatic event. Treatment of these teeth can range from no active treatment to tooth loss followed by prosthetic rehabilitation [8]. The prognosis of TDIs largely depends upon the actions taken at the time of injury [1]. Providing first aid management to the traumatized tooth (control of bleeding, cleaning and debridement of soft tissues, and positive reassurance) largely reduces anxiety of patients, minimizes bacterial invasion, ensures pulp, periodontal, and bony healing, and improves prognosis of traumatized tooth [8, 9]. Hence, parents, teachers, and other nondental personnel can play a crucial role by providing first‐aid management to traumatized teeth, which prevents long‐term complications and improves the prognosis of the injured teeth.

Moreover, studies have shown that there is limited or inadequate knowledge among the primary school teachers on the first aid management of TDIs [10–14]. However, there is a paucity of information of primary school teachers’ knowledge on TDIs in our part of the country. Hence, the study was aimed to determine the knowledge on first aid management of TDIs among primary school teachers in Dharan, Nepal.

## 2. Materials and Methods

### 2.1. Study Design and Ethical Approval

This cross‐sectional study was conducted from 7th November 2023 to15th February 2024 using the “Strengthening the Reporting of Observational Studies in Epidemiology (STROBE)” guideline [15]. The study adhered to the World Medical Association Declaration of Helsinki, and ethical clearance was obtained from Institutional Review Committee (IRC), BPKIHS, Dharan (Ref. No: 220/080/081‐IRC).

### 2.2. Setting, Participants, and Study Size

#### 2.2.1. Setting

This study was carried out among primary school teachers (Grade 1–5) of different schools (public and private) located in Dharan, Nepal.

#### 2.2.2. Eligibility Criteria

1. Inclusion criteria: all primary school teachers from Grade 1–5 of selected schools of Dharan city.
2. Exclusion criteria: those primary school teachers who did not give consent to participate in the study.

#### 2.2.3. Study Size

Sample size for this study was obtained considering 55% of the participants responded correctly in the management of TDI (avulsion) from the study done by Nirwan et al. [12] in South Jaipur, 2016. Considering 95% CI and permissible error of 10%, the final sample size obtained was 110 using the formula *n* = Z_α_^2^ pq/L^2^.

### 2.3. Data Collection Technique and Tools

#### 2.3.1. Data Collection Technique

Initially, out of total 20 wards in Dharan city of Nepal, four wards were selected randomly. Number of schools (both private and public) within that ward was selected randomly until the desired sample size was achieved. A total of 110 primary school teachers from both public and private schools from Grade 1 to Grade 5 were equally enrolled (public = 55 and private = 55) in this study. The purpose of taking equal number of public and private school teachers was for the comparative purpose. Permission from the selected schools and informed consent from all the participants were obtained. Standard translated and validated self‐administered questionnaires were distributed to the participants and instructed them to fill the form.

#### 2.3.2. Data Collection Tool

The questionnaire was adapted from previous studies on the knowledge of first aid management of TDIs among primary school teachers [11, 13]. The English questionnaire was translated and validated in Nepali language using standard translation method. A total of 12 questions were there in the questionnaire. The questionnaire was divided into two parts: Part I comprised personal/professional characteristics of the participants, and Part II comprised the questions in the management of TDIs, which had three major questions based on the clinical scenario with its sub‐questions.

Pretesting of the questionnaire was done on 10% of the participants to check if the participants clearly understood the questions being asked and their willingness to answer the question. Cronbach alpha obtained for the questionnaire was 0.89 indicating good agreement [16].

### 2.4. Statistical Analysis

Collected data were entered in Microsoft Excel Sheet Version 2016 and analyzed using Statistical Package for Social Sciences (SPSS Version 21.5). For descriptive statistics, mean, standard deviation, frequency, and proportion were used along with graphical presentation. Knowledge level was assessed by calculating the overall correct response percentage and categorizing as <40%, 40%–60%, 60%–80%, and >80% as having poor, average, good, and very good knowledge [17]. Chi‐square test was used for inferential statistics. Multiple logistic regression analysis was performed to see the effect of different predictors on knowledge of the participants. The level of significance was set at *p*  < 0.05.

## 3. Results

A total of 110 school teachers from both public and private schools were included in the study. Overall, the mean age of the participants was 36.38 ± 10.98 years, and the majority of them were females (73.6%). Of the total school teachers, only 26 (23.6%) were informed or trained about dental trauma, and they acquired information from different sources among which internet was the primary source of information in the management of TDIs (57.7%). More than half of the participants (57.7%) stated that they had witnessed dental trauma injuries during school hours (Table 1).

**Table 1:** Demographic and professional characteristics of the participants (*n* = 110).

Characteristics	*n* (%)
Personal characteristics	
Age (mean ± SD)	36.38 ± 10.98
Gender	
Male	29 (26.4)
Female	81 (73.6)
Professional characteristics	
Type of school	
Public	55 (50.0)
Private	55 (50.0)
Teaching experience	
<10 Years	55 (50.0)
>10 Years	55 (50.0)
Have you ever been trained or informed about dental trauma?	
Yes	26 (23.6)
No	84 (76.4)
If yes, how?	
During first aid course	7 (26.9)
Formal expert training/information	1 (3.8)
Information leaflets	3 (11.5)
Internet sources	15 (57.7)
Have you ever helped a child after dental injury at school?	
Yes	71 (64.5)
No	39 (35.5)
If yes, how many dental trauma injuries have you seen during school hours?	
1–2	41 (57.7)
3–4	17 (23.9)
>5	13 (18.3)
Do you believe in your ability to help a child with dental trauma injury?	
Yes	74 (67.3)
No	36 (32.7)

### 3.1. Knowledge on Management of Dental Trauma Cases

#### 3.1.1. Case I: Broken Incisor of 10‐YearOld Girl

Nearly half of the participants (49.1%) were not able to differentiate between the broken upper front teeth of a 10‐year‐old child that was temporary or permanent. Majority of them were unaware about its management (44.5%).

#### 3.1.2. Case II: Lateral Luxation

More than two‐third of the participants (75.5%) were unaware about the proper management of lateral luxation. Public school teachers had higher knowledge on its management (30.9%) as compared to private school teachers (16.4%).

#### 3.1.3. Case III: Avulsion

Of the total participants, only four of them (3.6%) responded correctly about the proper management of the avulsed tooth. More than two‐third of the participants (83.6%) were unaware about the replantation of the avulsed tooth. Only few of them (4.5%) had knowledge about the best storage media to save the avulsed tooth until the child visits the dentist.

The responses of the participants on the questionnaire related to dental trauma cases have been presented in Table 2.

**Table 2:** Knowledge of participants on dental trauma injury‐related questions (*n* = 110).

Questions	(*n* [%])		Total (*n* [%]) (*n* = 110)
	Public (*n* = 55)	Private (*n* = 55)	
Case I: During school hours, 10‐year‐old girl fell and upper two front teeth are broken.			

Q.1 The broken teeth are likely to be?			
(a) Temporary teeth	29 (52.7)	25 (45.5)	54 (49.1)
(b) Permanent teeth	20 (36.4)	24 (43.6)	44 (40.0)
(c) Don’t know	6 (10.9)	6 (10.9)	12 (10.9)
Q.2 What emergency management will you do in this case?			
(a) Calm down the child and send her back to the class.	2 (3.6)	1 (1.8)	3 (2.7)
(b) Contact parents and advise them to send child to the dentist immediately.	41 (74.5)	48 (87.3)	89 (80.9)
(c) Look for the broken tooth piece and send the child to the dentist with it.	12 (21.8)	6 (10.9)	18 (16.4)
Q.3 How should the broken piece be stored?			
(a) Dry environment	16 (29.1)	23 (41.8)	39 (35.5)
(b) Moist environment	11 (20.0)	11 (20.0)	22 (20.0)
(c) Don’t know	28 (50.9)	21 (38.2)	49 (44.5)

Case II: A 13‐year‐old boy is hit in the face due to which upper front teeth are moved laterally and bleeding is visible in the gums.			

Q.1 What emergency management will you do?			
(a) Calm down the child, rinse the area with plenty of water, and advise the child to bite on the gauze for bleeding control before contacting the parents and referring to dentist.	37 (67.3)	46 (83.6)	83 (75.5)
(b) Calm down the child, contact parents, and advise them to go immediately to dentist.	17 (30.9)	9 (16.4)	26 (23.6)
(c) Don’t know	1 (1.8)	0 (0.0)	1 (0.9)

Case III: A 15‐year‐old boy is hit in the face and two upper front teeth falls in the ground, there is blood in his mouth.			

Q.1 What emergency management will you do in this case?			
(a) Stop the bleeding by compressing a cloth over the injury.	34 (61.8)	46 (83.6)	80 (72.7)
(b) Look for the tooth, wash it, and put it back in its place.	3 (5.5)	1 (1.8)	4 (3.6)
(c) Place the tooth in child’s mouth and look for professional help.	12 (21.8)	1 (1.8)	13 (11.8)
(d) Place the tooth in a paper and send the child to dentist after the school time.	6 (10.9)	7 (12.7)	13 (11.8)
Q.2 Can the teeth be repositioned?			
(a) Yes	12 (21.8)	6 (10.9)	18 (16.4)
(b) No	43 (78.2)	49 (89.1)	92 (83.6)
Q.3 If yes, within which time limit can this be performed?			
(a) Immediately, within first 30 mins from the injury.	1 (1.8)	2 (3.6)	3 (2.7)
(b) Within 1–5 h after the injury.	4 (7.3)	0 (0.0)	4 (3.6)
(c) Within first 48 h after the injury.	1 (1.8)	0 (0.0)	1 (0.9)
(d) There is no time limit restriction.	1 (1.8)	2 (3.6)	3 (2.7)
(e) Don’t know	48 (87.3)	51 (92.7)	99 (90.0)
Q.4 If the tooth has fallen on the dirty ground, what would you do?			
(a) Rinse the tooth under tap water and put it back into its socket.	9 (16.4)	7 (12.7)	16 (14.5)
(b) Rub away the dirt by a sponge and soap and put it back in its place.	16 (29.1)	4 (7.3)	20 (18.2)
(c) Put it back into the socket immediately without cleaning.	1 (1.8)	0 (0.0)	1 (0.9)
(d) Discard the tooth.	29 (52.7)	44 (80.0)	73 (66.4)
Q.5 How would you store the teeth until the child visits dentist?			
(a) In ice	6 (10.9)	6 (10.9)	12 (10.9)
(b) In child’s mouth	5 (9.1)	0 (0.0)	5 (4.5)
(c) In child’s hand	0 (0.0)	0 (0.0)	0 (0.0)
(d) Wrap the teeth in handkerchief or paper tissue	35 (63.6)	43 (78.2)	78 (70.9)
(e) Any other^a^	9 (16.4)	6 (10.9)	15 (13.6)
After a dental trauma injury which type of health service would you seek first?			
(a) General physician	13 (23.6)	4 (7.3)	17 (15.4)
(b) Hospital	6 (10.9)	0 (0.0)	6 (5.5)
(c) Nearest general dentist	36 (65.5)	51 (92.7)	87 (79.1)

^a^Any other: Save the tooth by immersing in bowl of water, don’t know.

**Figure 1:**
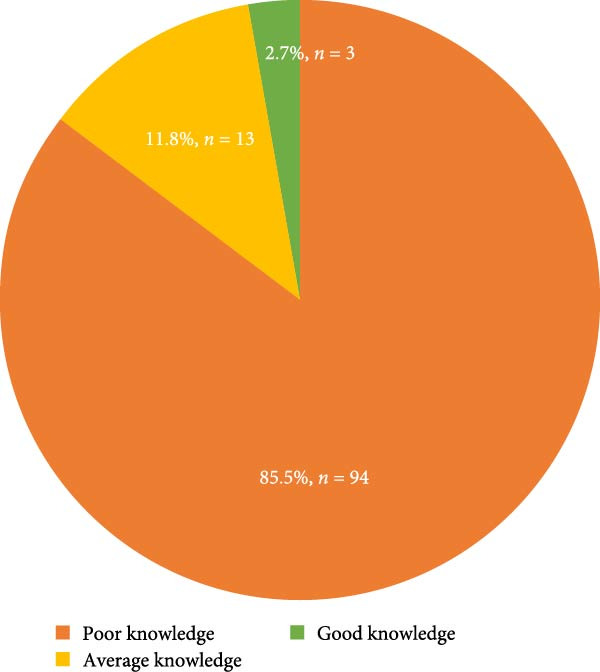
Overall distribution of knowledge in the management of traumatic dental injuries among participants (*n* = 110).

Overall, majority of the participants, 94 (85.5%), had poor knowledge on the primary management of TDI to children at schools. Only three school teachers (2.7%) had good knowledge on the same (Figure 1).

No significant difference was observed between knowledge and gender, years of experience, and type of school regarding dental trauma management (Table 3). A significant difference was observed in the primary management of avulsed tooth between teachers of public and private schools (*p* = 0.007) (Table 4).

**Table 3:** Association of knowledge with participant’s characteristics (*n* = 110).

Gender	Knowledge (*n* [%])			
	Good	Average	Poor	*p*‐Value
Male	1 (3.4)	5 (17.2)	23 (79.3)	0.541^a^
Female	2 (2.5)	8 (9.9)	71 (87.7)	
Years of experience				
Less than 10	1 (1.8)	5 (9.1)	49 (89.1)	0.550^a^
More than 10	2 (3.6)	8 (14.5)	45 (81.8)	
Type of schools				
Public	2 (3.6)	7 (12.7)	46 (83.6)	0.797^a^
Private	1 (1.8)	6 (10.9)	48 (87.3)	

^a^Chi‐square test.

**Table 4:** Association of type of school and management of traumatic dental injuries (*n* = 110).

Dental trauma cases	Correct responses (*n* [%])		*p*‐Value
	Public	Private	
Broken tooth management	12 (21.8)	6 (10.9)	0.236^a^
Lateral luxation management	17 (30.9)	9 (16.4)	0.109^a^
Avulsion management	3 (5.5)	1 (1.8)	0.007^a^

^a^Chi‐square test.

On computing multiple logistic regression analysis to see the predictors of adequate vs. poor knowledge, none of the variables were found to be statistically significant (Table 5).

**Table 5:** Multiple logistic regression analysis to see the predictors of knowledge.

Variables	β‐Coefficient	S.E.	95% CI (lower–upper)	*p*‐Value
Age	0.98	0.04	0.90–1.06	0.55
Gender				
Male (ref)	1.25	0.66	0.34–4.56	0.73
Female				
Type of school				
Public (ref)	1.07	0.67	0.29–3.97	0.92
Private				
Teaching experience				
<10 Years (ref)	0.81	0.83	0.16–4.16	0.80
>10 Years				
Training received				
Yes (ref)	0.62	0.82	0.12–3.07	0.55
No				

Abbreviation: ref, reference.

## 4. Discussion

The present cross‐sectional study assessed the knowledge on emergency management of TDIs among primary school teachers of Dharan, Nepal. The mean age of the participants was 36.38 ± 10.98 years, and the majority of them were females (73.6%). This is concordance to the findings of several other studies reported in Nepal [10], India [12], Greece [11], and Jordan [18] where higher percentage of female teachers participated.

The age and teaching experience of school teachers are considered as an important factor in deciding the knowledge level of participants. Interestingly, our present study found that there were equal number of teachers with teaching experience of more than or less than 10 years. In our present study, no significant association was observed between years of teaching experience and knowledge level of participants. This finding corroborates to the study done in India [12].

The current study found that only 23.6% of the total teachers were trained or informed about dental trauma. Similar finding was reported by Tzimpoulas et al. [11] where only 26.1% of the primary school teachers had information on dental trauma. Among those who reported to have known about TDIs, primary source of information for them was internet. This could be due to the easy access of internet where people use different social medias almost on a daily basis and can be utilized to look upon any health‐related information [19, 20].

About 27.0% of the participants had received first aid course training in the primary management of dental trauma cases. This is higher than reported by Nirwan et al. [12] (12.5%), Tzimpoulas et al. [11] (9%), Tahririan et al. [17] (10.9%), and Al‐Obaida [14] (17.8%). However, in contrary to our present study, Pradhan et al. [10] (31.6%), AI‐Jundi et al. [18] (46%), and Dauparė and Narbutaite [13] (99.1%) reported higher percentage of participants receiving first aid course in dental trauma management. It is important to train school teachers periodically through first aid course on emergency management of TDIs since teachers are the primary people to provide immediate treatment to children on dental emergency during school hours [18].

More than half of the participants (64.5%) in the present study reported that they had helped a child after dental trauma at school and maximum number of dental trauma cases witnessed was one or two. This is contrary to the study done in Lithuania [13] where only 43.3% of the school teachers had helped children with dental trauma occurring at school. Two‐third (67.3%) of the primary school teachers stated that they had the ability to help children after dental trauma injury, which is higher than that reported in Greece [11] and lower than that reported in Jordan [18] with 66% and 85.8%, respectively.

In regard to dental trauma injury‐related questions, only 40.0% of the participants knew that the broken front teeth of 10‐year‐old girl was permanent teeth. This is comparable to the finding reported by Raoof et al. [21] and higher than that reported by Al‐Obaida. [14] and Mohandas and Chandan [22]. However, Dauparė and Narbutaite [13] and Tzimpoulas et al. [11] reported that higher percentage of school teachers were able to distinguish between primary or permanent tooth that was broken. The lower percentage obtained in the present study could be due to inadequate general dental knowledge among primary school teachers as reported by AI‐Jundi et al. [18]. It is important that the school teachers should be able to differentiate between primary and permanent tooth that has suffered dental trauma as it helps in determining the emergency treatment and long‐term prognosis of the fractured tooth [12].

Only few participants (16.4%) stated that they would look for the fractured tooth piece and send the child to dentist with it, which is concordance to the finding reported by Dauparė and Narbutaite [13] but contrary to the finding reported by Tzimpoulas et al. [11] and Raoof et al. [21]. It is necessary to inform school teachers about the reattachment of broken tooth fragment.

Majority of the participants were unaware about the proper storage of broken tooth piece, and only 20% knew that it should be stored in moist environment, which is lower than that reported in Greece [11].

In the second part of the question regarding the primary management of lateral luxation, more than two‐third of the participants (75.5%) responded that the control of bleeding is immediate management of the displaced tooth. Although bleeding is a clinical consequence of an injury and it is an uncomfortable situation [11], it should be understood that applying pressure to the displaced teeth (biting gauze piece to control bleeding) can do more harm than doing good to the injured tooth. Hence, emergency management in this case should be immediate referral of child to the dentist after contacting parents. This was reported by 23.6% of the participants in the present study. In contrary to this, Tzimpoulas et al. [11] found higher percentage of participants (69.9%) reporting immediate referral of child to dentist as the emergency management of lateral luxation, whereas Nirwan et al. [12] reported that 31% of the participants knows the correct management of displaced teeth.

In the third part of the question related to the primary management of avulsed teeth, it was found that majority of the participants (72.7%) concentrated on stoppage of bleeding after tooth avulsion and very few (3.6%) of them reported relocating of the avulsed tooth back in its place as its primary management. This could be due to unawareness among primary school teachers about the replantation of the avulsed tooth back to its own place as its immediate management [12]. Also, majority of the people think that bleeding is dangerous and is an uncomfortable situation, which makes them more focused in control of bleeding [17]. Similar result was reported by the study done in Panevezys, Lithuania [13]. In contrary to this, higher percentage of Iranian school teachers [21] stated putting back avulsed tooth in its place, and 56.8% of the school teachers in Jaipur [12] stated that they would try to relocate an avulsed tooth.

More than two‐third of the participants (83.6%) were unaware about the repositioning of the avulsed tooth and the time limit of its replantation. Only 2.7% of the participants responded correctly that it should be performed immediately within first 30 min after the injury. This is contrary to the finding reported in Greece [11] and Bangalore [22] where 52.2% and 22% of the participants knew that avulsed tooth can be replanted and this should be done immediately within first 30 min. Time lapse between the injury and treatment of the avulsed tooth is an important factor in determining its prognosis since shorter extra oral time results in more favorable replantation outcomes.

Storage media is another important factor that determines the viability and outcome of the avulsed tooth. Best storage media should be such that it should be able to preserve cell vitality until tooth replantation and should be readily available at the site of injury [19]. Cold milk or saliva (child’s vestibule) is considered to be the best storage media for avulsed tooth as it delays the death of periodontal ligament cells and preserves cell viability [23]. However, only 4.5% of the total participants in the present study correctly responded that child’s mouth is the appropriate storage media for avulsed tooth. Knowledge regarding the proper storage media of avulsed teeth was found to be limited in several other studies reported [11–14, 19, 24].

The present study showed that majority of the participants were not aware about the proper management of avulsed tooth that has fallen on the dirty ground as two‐third of them (66.4%) responded that the tooth should be discarded. Only 14.5% stated that it should be rinsed in tap water and put back in its socket. This is contrary to the finding reported in India [12], Lithuania [13], Saudi Arabia [14], Iran [21], and Bangalore [22] where higher percentage of the participants responded correctly about the management of avulsed tooth that has fallen in the dirty ground.

Majority of the participants (79.1%) in the present study stated that the nearest general dentists should be contacted immediately in case of any TDIs to children at school. This is in fact a good sign indicating an increased recognition of dentists’ role in the community where children are sent to correct places for the treatment of dental injuries without wasting their time, which improves the prognosis of the injured tooth. This is concordance to the findings reported in Brazil [24] and Greece [11].

Our present study found that public school teachers had comparatively good knowledge than private school teachers in the management of TDIs, but this difference was not statistically significant. However, in the management of avulsed tooth, difference between public and private school teachers’ knowledge was found to be statistically significant. This could be due to more number of public school teachers being informed or trained about primary management of dental trauma cases at schools and also due to increased dental trauma cases witnessed at schools, which improved their knowledge in the primary management of avulsed tooth.

Overall, this present study found that majority of the participants (85.5%) had poor knowledge in the primary management of TDIs at school, which corroborates to the finding reported in India [12]. This could be due to the limited information or training provided to the primary school teachers on emergency management of TDIs.

Teachers’ knowledge in the management of TDIs can be improved by providing them the required yearly first aid training classes. Integrating first aid module in the teachers training curriculum can improve their knowledge in the management of TDIs. Beside this, other way to increase their understanding of dental trauma management includes implementing nationwide awareness campaigns, education interventions and campaigns that include posters in schools, and lectures that use audiovisual aids to increase teachers’ understanding of dental trauma management. To create suitable, practical continuing dental education programs, dentist and educators must work together [11, 25, 26].

As strengths of this study, we highlight that this is the first study in eastern Nepal carried out to assess the knowledge among primary school teachers in the first aid management of TDIs. Equal number of public and private school teachers were included in the present study to facilitate the comparison of knowledge level in between them. Randomly selected wards and schools in this study increased the generalizability of the study among primary school teachers of Dharan city.

However, this study has some limitations. Since this is a self‐reported questionnaire‐based study, there are chances of recall bias in it. This study involves enrollment of school teachers from a single city, which limits the generalizability of the study to the school teachers of other places of Nepal.

Overall, the present study provides a baseline data, which can be utilized in conducting the further research nationwide and with other study designs. This would help in planning the strategies at national level to increase the knowledge among primary school teachers in the management of TDIs that would ultimately reduce the burden and complications from TDIs.

## 5. Conclusion

The findings of this study concluded that primary school teachers of Dharan, Nepal had poor knowledge in the first aid management of TDIs, which reflects a need to improve their knowledge on the same.

## Disclosure

All authors have read and approved the manuscript.

## Conflicts of Interest

The authors declare no conflicts of interest.

## Author Contributions

Ashma Ojha was involved in data collection and preparation of the manuscript. Ashish Shrestha and Tarakant Bhagat supervised, revised, and contributed to the preparation of manuscript. Santosh Kumari Agrawal helped in statistical analysis, reviewing, and editing of article.

## Funding

No funding was received for this research.

## Data Availability

The data that support the findings of this study are available from the corresponding author upon reasonable request.
